# Supplementary material for: Tyrosine phosphorylation regulates hnRNPA2 granule protein partitioning and reduces neurodegeneration
Source: EMBO J. 2020 Dec 22;40(3):e105001. doi: 10.15252/embj.2020105001 (PMC7849316; doi:10.15252/embj.2020105001)
Supplement: Supplementary file 1 — Appendix [file EMBJ-40-e105001-s001.pdf]

Appendix Figures:

Tyrosine phosphorylation regulates hnRNPA2 granule protein partitioning & reduces neurodegeneration

Veronica H. Ryan[1], Theodora Myrto Perdikari[2], Mandar T. Naik[3], Camillo F. Saueressig[4], Jeremy Lins[4], Gregory L. Dignon[5], Jeetain Mittal[5], Anne C. Hart[4], Nicolas L. Fawzi[3]

[1]Neuroscience Graduate Program, [2]Biomedical Engineering Graduate Program, [3]Department of Molecular Pharmacology, Physiology, and Biotechnology, [4]Department of Neuroscience, Brown University, Providence, RI 02912, USA

[5]Department of Chemical and Biomolecular Engineering, Lehigh University, Bethlehem, PA 18015, USA.

Correspondence: Nicolas L. Fawzi (nicolas_fawzi@brown.edu) and Anne C. Hart (anne_hart@brown.edu)

Keywords: Fyn/hnRNPA2/liquid-liquid phase separation/neurodegeneration/tyrosine phosphorylation

**Table of Contents**	**Page Number**
Appendix Figure S1	2
Appendix Figure S2	3
Appendix Figure S3	4
Appendix Figure S4	6
Appendix Figure S5	7
Appendix Figure S6	8
Appendix Figure S7	10
Appendix Figure S8	12
Appendix Figure S9	14
Appendix Figure S10	15
Appendix Figure S11	16
Appendix Figure S12	18
Appendix Table S1	19
Appendix Table S2	24

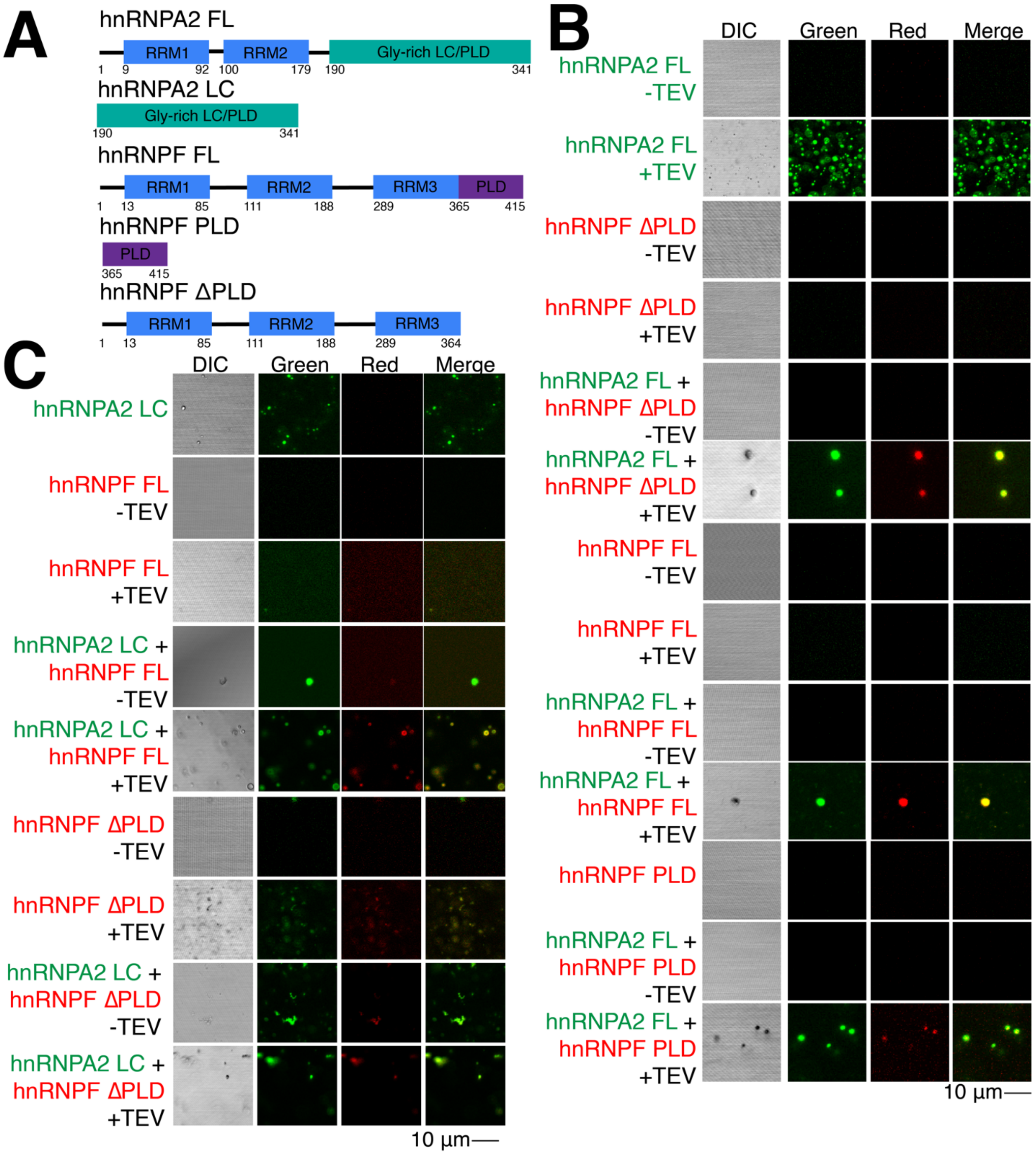

Appendix Figure S1: hnRNPF partitions into hnRNPA2 LC droplets. Related to Figure 1.

A) Schematic of protein constructs used in this figure.

B) After cleavage of an N-terminal maltose binding protein solubility tag, hnRNPF FL and hnRNPF ΔPLD do not undergo LLPS. Each one can partition into hnRNPA2 FL droplets. hnRNPA2 FL droplets appear after cleavage of the C-terminal maltose binding protein solubility tag. Conditions: 20 µM proteins (~1% fluorescently tagged), 20 mM Tris pH 7.4 50 mM NaCl. Scale bar: 10 µm

C) After cleavage of an N-terminal maltose binding protein solubility tag, hnRNPF FL can partition into hnRNPA2 LC droplets. Conditions: 20 µM proteins (~1% fluorescently tagged), 20 mM MES pH 5.5 50 mM NaCl, 150 mM urea. Scale bar: 10 µm

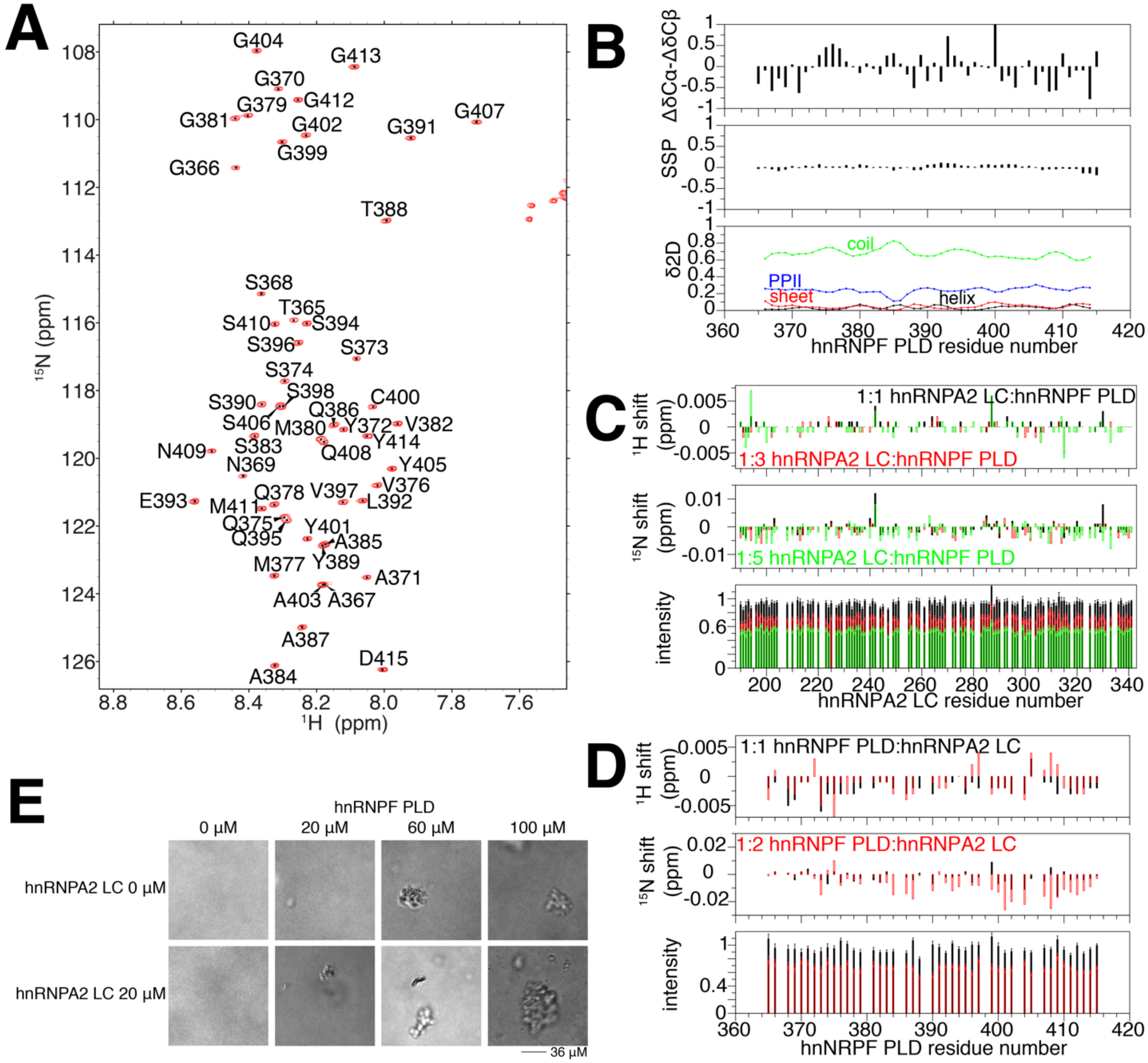

Appendix Figure S2: hnRNPF PLD is disordered and induces aggregation of hnRNPA2 LC. Related to Figure 1.

A) ^{1}H-^{15}N HSQC of hnRNPF PLD is consistent with intrinsic disorder. Conditions: 150 µM hnRNPF PLD, 20 mM MES pH 5.5, 1 mM DTT, 298K.

B) ΔδCα-ΔδCβ, SSP, and δ2D show hnRNPF PLD is predominantly disordered.

C) Titration of natural isotopic abundance (n.a.) hnRNPF PLD into ^{15}N hnRNPA2 LC results in small chemical shifts but decreasing signal intensity with increasing hnRNPF PLD, consistent induction of LLPS or aggregation. Conditions: 20 µM hnRNPA2 LC, varying hnRNPF PLD concentration, 20 mM MES pH 5.5, 150 mM urea, 1 mM DTT, 298K.

D) Titration of natural isotopic abundance (n.a.) hnRNPA2 LC into ^{15}N hnRNPF PLD results in small chemical shifts but decreasing signal intensity with increasing hnRNPA2 LC, consistent with induction of LLPS or aggregation. Conditions: 20 µM hnRNPF PLD, varying hnRNPA2 LC concentration, 20 mM MES pH 5.5, 150 mM urea, 1 mM DTT, 298K.

E) DIC micrographs show that hnRNPF PLD aggregates at higher concentrations in pH 5.5 MES and induces hnRNPA2 LC aggregation when mixed. Conditions: protein concertation as indicated, 20 mM MES pH 5.5, 0 mM NaCl 150 mM urea, 298K. Scale bar 36 µm.

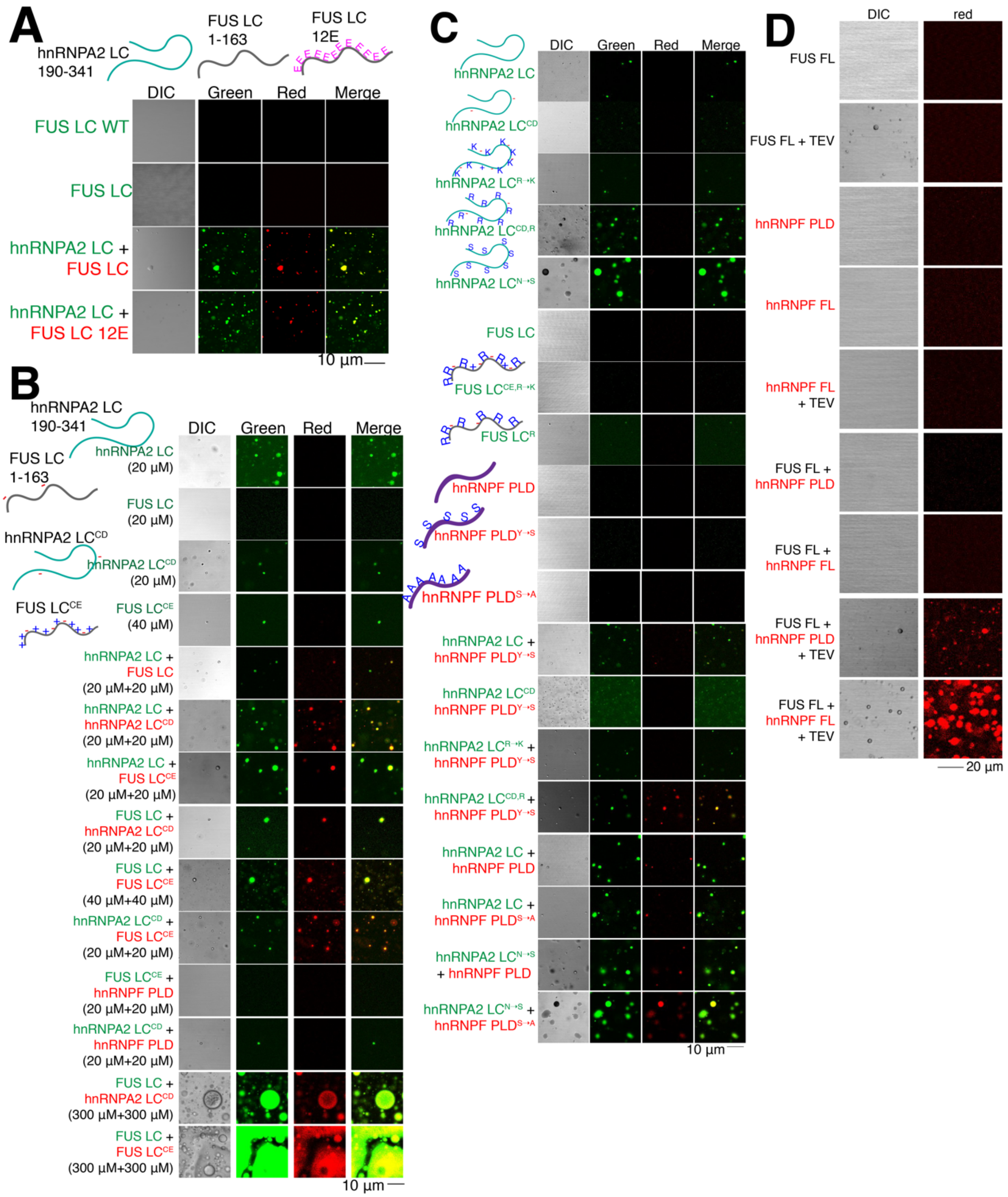

Appendix Figure S3: hnRNPF PLD WT and mutants partition into hnRNPA2 LC droplets. Related to Figure 1.

A) FUS LC WT and phosphomimic 12E (Monahan et al., 2017) can partition into hnRNPA2 LC droplets. Conditions: 20 µM proteins, 20 mM MES pH 5.5 50 mM NaCl. Scale bar: 10 µm

B) Brightfield and fluorescence micrographs of control experimental conditions for Figure 1D. Protein concentrations indicated next to images. Scale bar: 10 µm.

C) Brightfield and fluorescence micrographs of control experiments for Figure 1E. hnRNPF PLD$^{Y\rightarrow S}$ and hnRNPF PLD$^{S\rightarrow A}$ mutations have no effect on partitioning. FUS LC$^{CE,R\rightarrow K}$ (FUS LC with hnRNPA2 LC-like charge with all arginine changed to lysine) is unable to undergo LLPS at 20 μM, as is FUS LC with arginines introduced (FUS LC^{R}). Changing the asparagines of hnRNPA2 to serine (hnRNPA2 LC$^{N\rightarrow S}$) does not alter partitioning of hnRNPF PLD or hnRNPF PLD$^{S\rightarrow A}$, indicating that not all residue substitutions interfere with hnRNPF PLD partitioning into hnRNPA2 LC. Conditions: 20 μM proteins unless otherwise indicated (~1% fluorescently tagged), 20 mM MES pH 5.5 50 mM NaCl. Scale bar: 10 μm.
D) hnRNPF PLD and hnRNPF FL (AlexaFluor555) are both able to partition into FUS FL droplets, likely due to interactions with the FUS RGG domains. Conditions: 10 μM proteins (~1% fluorescently tagged hnRNPF), 20 mM Tris pH 7.4, 150 mM NaCl. Scale bar 20 μm.

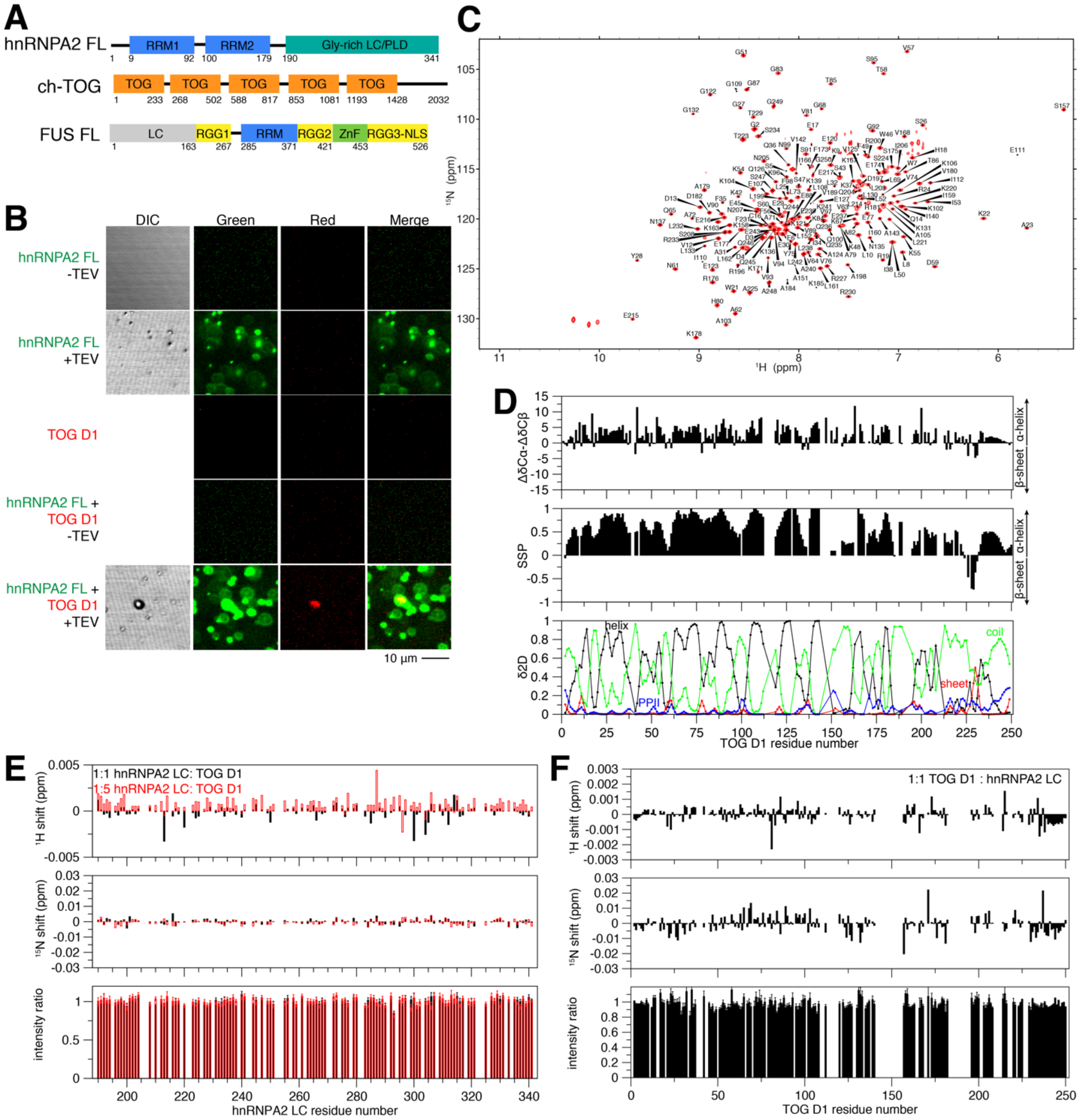

Appendix Figure S4: TOG D1 partitions into hnRNPA2 LC and FUS FL droplets. See Fig 2.

A) Domain structure of hnRNPA2, TOG, and FUS proteins.

B) TOG D1 can partition into hnRNPA2 FL droplets. Conditions: 20 µM proteins (~1% fluorescently tagged), 20 mM Tris pH 7.4 50 mM NaCl. Scale bar: 10 µm

C) ^{1}H-^{15}N TROSY of ^{2}H ^{15}N TOG D1 is consistent with a protein with high α-helical content. Conditions: 1.4 mM protein, 20 mM MES pH 5.5, 298K.

D) ΔδCα-ΔδCβ, SSP, and δ2D indicate that TOG D1 is a globular protein composed mostly of α-helices and disordered linkers.

E-F) Titrations of **(E)** natural isotopic abundance (n.a.) TOG D1 with ^{15}N hnRNPA2 LC and **(F)** natural isotopic abundance (n.a.) hnRNPA2 LC into ^{2}H ^{15}N TOG D1 results in small chemical shifts and no change in signal intensity, indicating a very weak interaction. Conditions: 20 µM ^{15}N hnRNPA2 LC, 20 mM MES pH 5.5 150 mM urea, 298K.

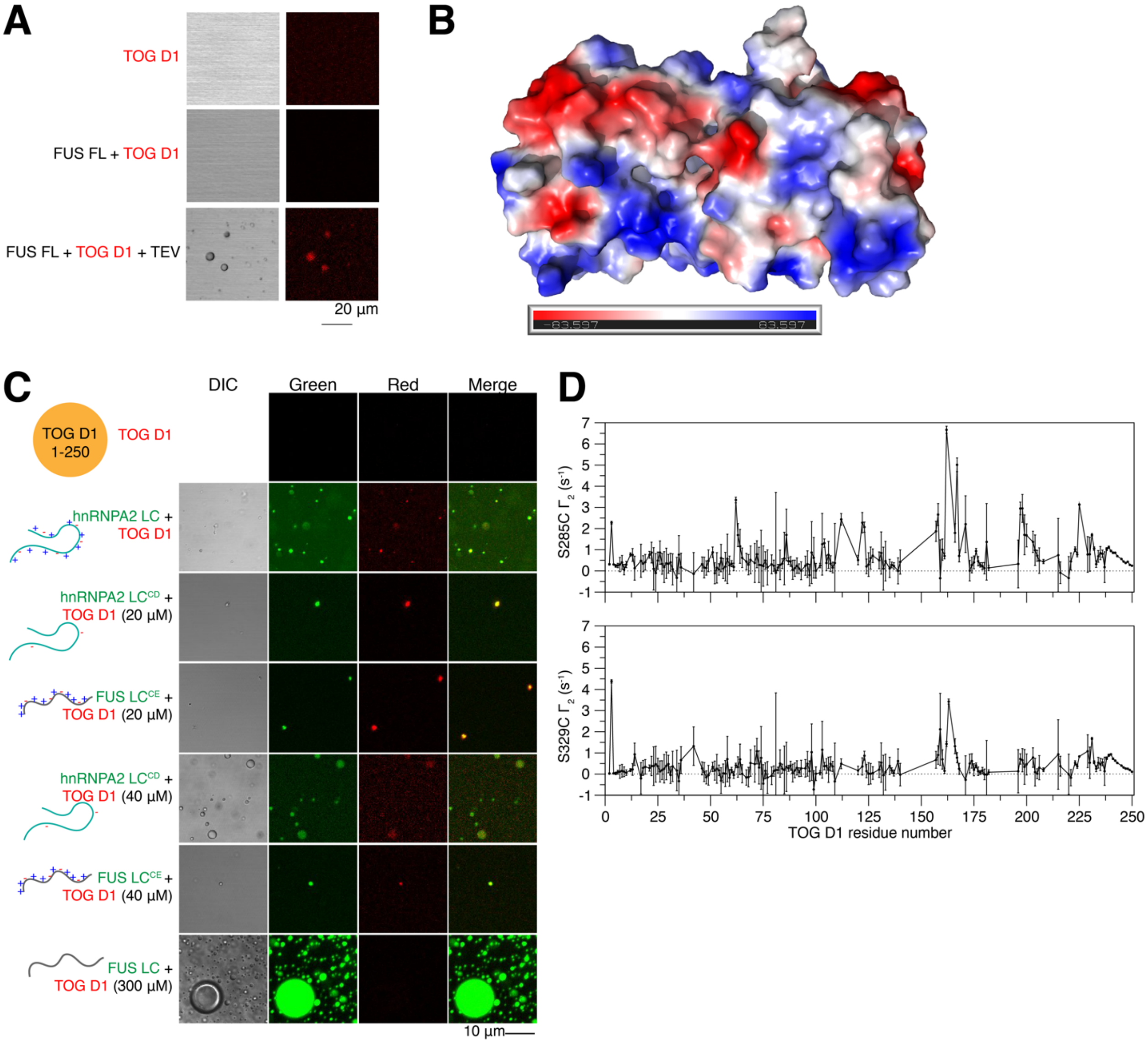

Appendix Figure S5: TOG D1 interacts with hnRNPA2 LC weakly. Related to Figure 2.
A) TOG D1 partitions into FUS FL droplets. Experiment run concurrently with experiments in S3D, see there for FUS FL control images. Conditions: 10 µM proteins (~1% fluorescently tagged hnRNPF), 20 mM Tris pH 7.4, 150 mM NaCl. Scale bar 20 µm.
B) TOG D1 homology structure with surface charge indicates that the surface of TOG D1 is highly charged.
C) hnRNPA2 LC^{CD} and FUS LC^{CE} do not alter partitioning of TOG D1. Conditions: 20 µM hnRNPA2 LC and hnRNPA2 LC^{CD}, 40 µM FUS LC^{CE}, TOG D1 concentration matches other protein in mixture (either 20 or 40 µM) (all ~1% fluorescently tagged), 20 mM MES pH 5.5 50 mM NaCl, 150 mM urea.
D) Quantification of PRE experiments in Figure 2C-D. PREs are overall weaker with hnRNPA2 LC S329C than with hnRNPA2 LC S285C, suggesting that the region around 285 of hnRNPA2 LC interacts with TOG D1 more. Conditions: 250 µM ^{2}H ^{15}N TOG D1, 50 µM natural abundance hnRNPA2 LC-MTSL, 20 mM MES pH 5.5 150 mM urea, 298K. Error bars are confidence interval corresponding to standard deviation derived from parameter best fit (6 data points).

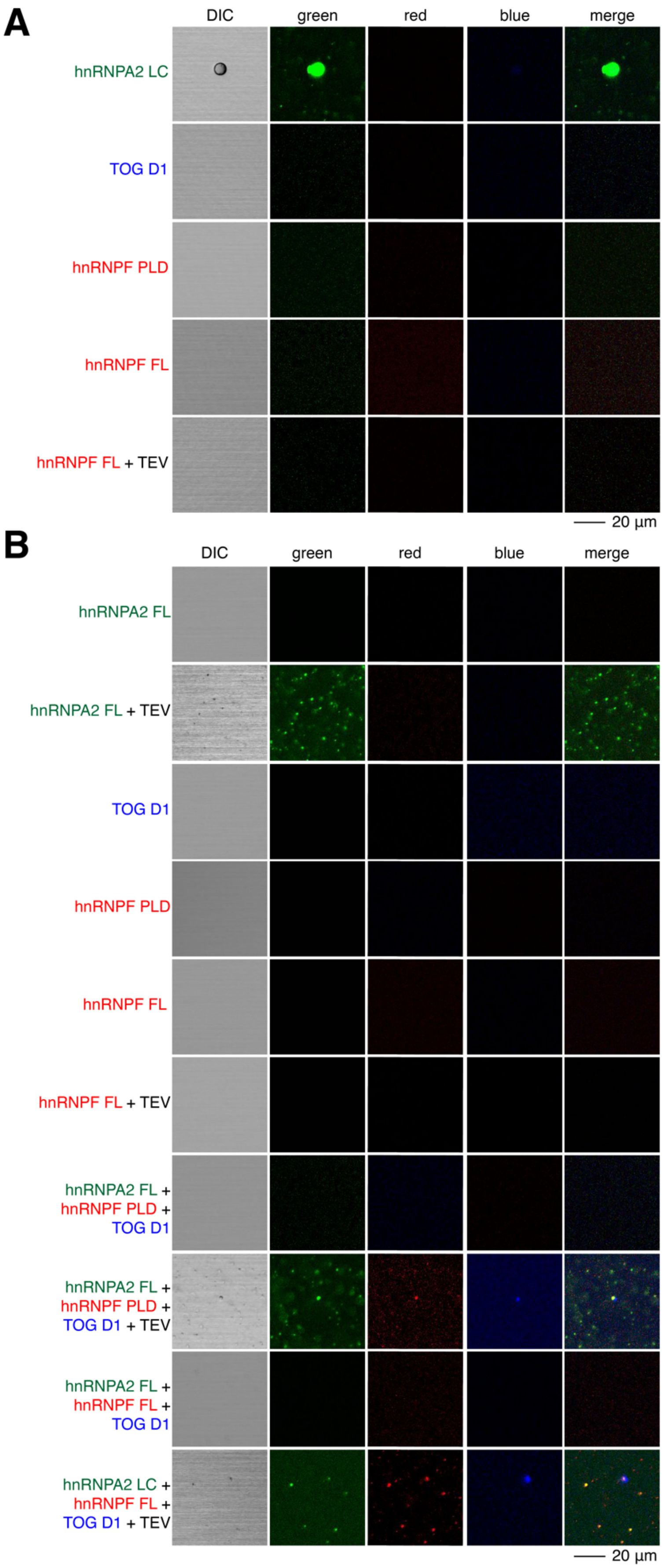
A
DIC
green
red
blue
merge
hnRNPA2 LC
TOG D1
hnRNPF PLD
hnRNPF FL
hnRNPF FL + TEV
20 µm
B
DIC
green
red
blue
merge
hnRNPA2 FL
hnRNPA2 FL + TEV
TOG D1
hnRNPF PLD
hnRNPF FL
hnRNPF FL + TEV
hnRNPA2 FL + hnRNPF PLD + TOG D1
hnRNPA2 FL + hnRNPF PLD + TOG D1 + TEV
hnRNPA2 FL + hnRNPF FL + TOG D1
hnRNPA2 LC + hnRNPF FL + TOG D1 + TEV
20 µm

Appendix Figure S6: hnRNPF and TOG D1 can co-partition into hnRNPA2 droplets.
Related to Figure 2.

A) Single protein component controls for Fig 2E showing that only hnRNPA2 LC can phase separate on its own and supporting the observation that hnRNPF PLD and TOG D1 (and hnRNPF FL and TOG D1) are able to partition into hnRNPA2 LC droplets simultaneously. Conditions: 20 μM protein (~1% fluorescently tagged, hnRNPF FL is 10 μM), 20 mM MES pH 5.5 50 mM NaCl 150 mM urea. Scale bar: 20 μm.

B) hnRNPF PLD and TOG D1 (and hnRNPF FL and TOG D1) are able to partition into hnRNPA2 FL droplets simultaneously. Conditions: 20 μM protein (~1% fluorescently tagged, hnRNPF FL is 10 μM), 20 mM Tris pH 7.4 50 mM NaCl. Scale bar: 20 μm.

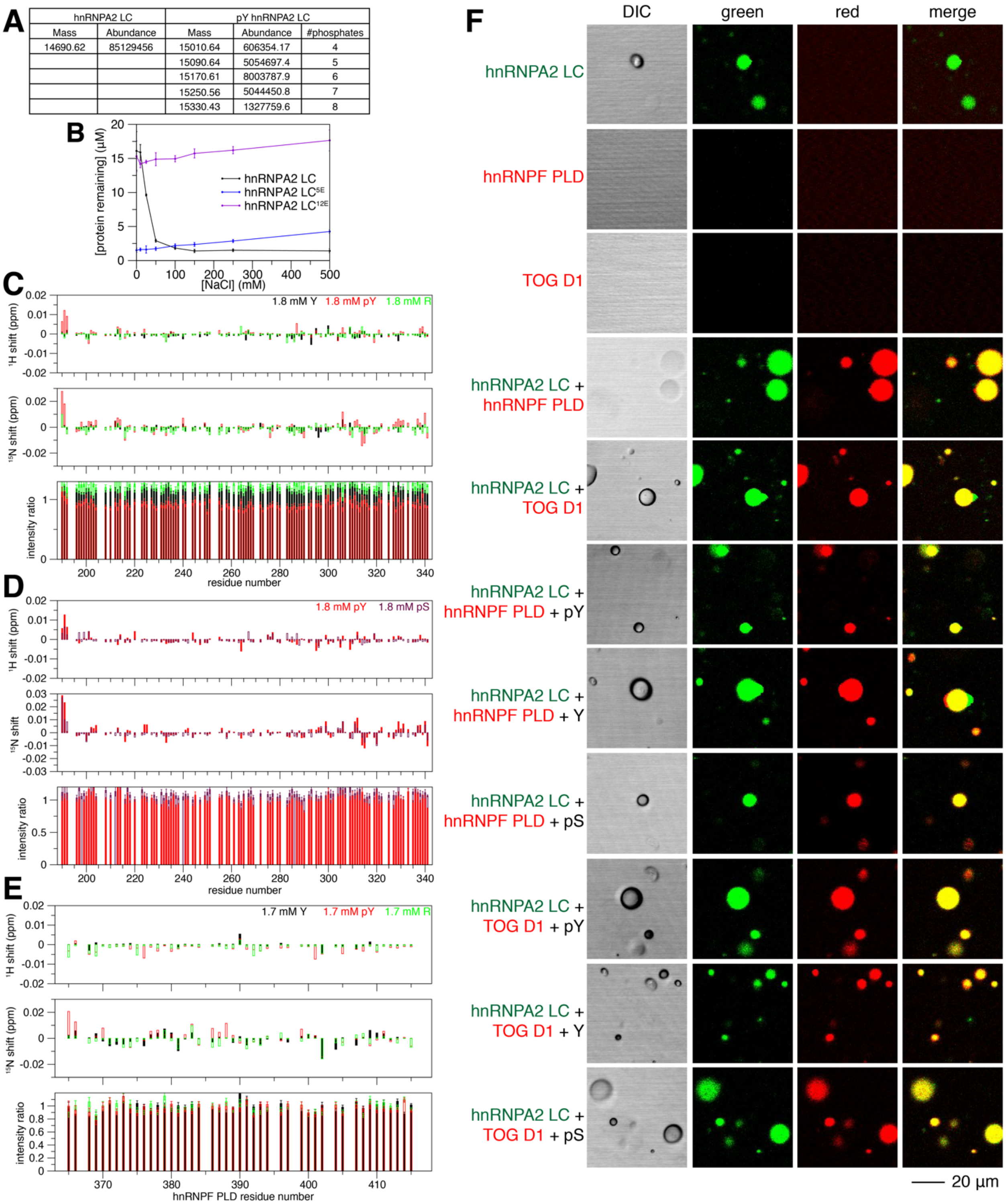

hnRNPA2 LC		pY hnRNPA2 LC		
Mass	Abundance	Mass	Abundance	#phosphates
14690.62	85129456	15010.64	606354.17	4
		15090.64	5054697.4	5
		15170.61	8003787.9	6
		15250.56	5044450.8	7
		15330.43	1327759.6	8

Appendix Figure S7: Phosphotyrosine interacts with arginines in hnRNPA2 LC. Related to Figure 3.

A) Mass spectrometry results showing a range of 4-8 phosphoryl groups have been added to hnRNPA2 LC, with six phosphorylation events being the most abundant.

B) Quantification of phase separation by spinning down phase separated droplets and measuring protein concentration remaining in the supernatant as a function of salt concentration. hnRNPA2

LC with 5 serine to glutamate phosphomimetic mutations (hnRNPA2 LC^{5E}, blue) is highly phase separated but phase separation slightly reduces with increasing salt concentration. hnRNPA2 LC with 12 serine to glutamate phosphomimetic mutations (hnRNPA2 LC^{12E}, purple) does not phase separate at any salt condition tested. Conditions: 20 µM protein, 20 mM MES pH 5.5, 150 mM urea, NaCl concentration as indicated, 25° C. Error bars are standard deviation of three replicates.
C) Titration of free tyrosine, phosphotyrosine, phosphoserine, or arginine with ^{15}N hnRNPA2 LC. Tyrosine, phosphoserine, and arginine all show small chemical shift perturbations and no intensity changes. In contrast, phosphotyrosine shows larger chemical shifts, particularly in regions of hnRNPA2 LC near arginine residues and shows a decrease in signal intensity, indicative of inducing LLPS at these low salt conditions. Conditions: 20 µM ^{15}N hnRNPA2 LC, 1.8 mM free amino acid, 20 mM MES pH 5.5 150 mM urea, 298K.
D) Titration of free phosphoserine and phosphotyrosine with ^{15}N hnRNPA2 LC. Phosphoserine shifts are smaller than those of phosphotyrosine and phosphoserine does not induce LLPS of hnRNPA2 LC. Conditions: 20 µM ^{15}N hnRNPA2 LC, 1.8 mM free amino acid, 20 mM MES pH 5.5 150 mM urea, 298K.
E) Titration of tyrosine, phosphotyrosine, and arginine with ^{15}N hnRNPF PLD. hnRNPF PLD does not phase separate in the presence of any of these amino acids and the chemical shifts are smaller than those of hnRNPA2 LC (see B), but phosphotyrosine induces the largest chemical shifts. Conditions: 20 µM ^{15}N hnRNPA2 LC, 1.8 mM free amino acid, 20 mM MES pH 5.5 150 mM urea, 298K.
F) Presence of 1.8 mM free tyrosine, phosphotyrosine, or phosposerine does not alter partitioning of hnRNPF PLD or TOG D1 into hnRNPA2 LC droplets. Conditions: 20 µM hnRNPA2 LC, 1.8 mM free amino acid, 20 mM MES pH 5.5, 50 mM NaCl, 150 mM urea, 298K.

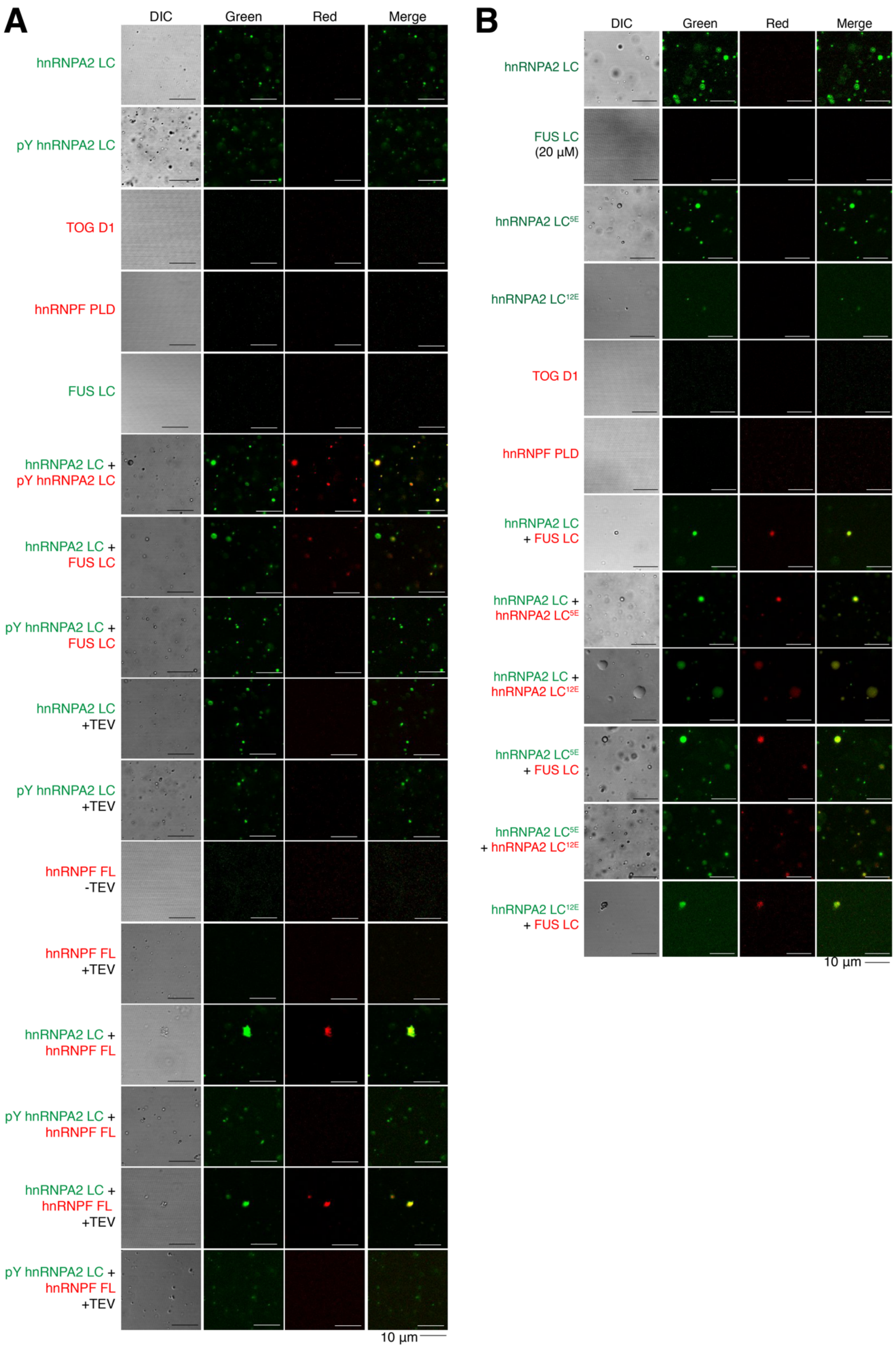
A
DIC
Green
Red
Merge
hnRNPA2 LC
pY hnRNPA2 LC
TOG D1
hnRNPF PLD
FUS LC
hnRNPA2 LC + pY hnRNPA2 LC
hnRNPA2 LC + FUS LC
pY hnRNPA2 LC + FUS LC
hnRNPA2 LC +TEV
pY hnRNPA2 LC +TEV
hnRNPF FL -TEV
hnRNPF FL +TEV
hnRNPA2 LC + hnRNPF FL
pY hnRNPA2 LC + hnRNPF FL
hnRNPA2 LC + hnRNPF FL +TEV
pY hnRNPA2 LC + hnRNPF FL +TEV
10 µm
B
DIC
Green
Red
Merge
hnRNPA2 LC
FUS LC (20 µM)
hnRNPA2 LC5E
hnRNPA2 LC12E
TOG D1
hnRNPF PLD
hnRNPA2 LC + FUS LC
hnRNPA2 LC + hnRNPA2 LC5E
hnRNPA2 LC + hnRNPA2 LC12E
hnRNPA2 LC5E + FUS LC
hnRNPA2 LC5E + hnRNPA2 LC12E
hnRNPA2 LC12E + FUS LC
10 µm

Appendix Figure S8: Phosphotyrosine modifications but not serine phosphomimics alter partitioning of hnRNPF PLD and TOG D1. Related to Figure 3.

A) Fluorescence micrographs of control experiments related to Figure 3C and partitioning of hnRNPF FL to hnRNPA2 LC or pY hnRNPA2 LC droplets. hnRNPF FL is also excluded from tyrosine phosphorylated hnRNPA2 LC. Conditions: 20 μM proteins (~1% fluorescently tagged), 20 mM MES pH 5.5, 50 mM NaCl, 150 mM urea.

B) Fluorescence micrographs of control experiments related to Figure 3D. Serine phosphomimic constructs are able to co-phase separate with WT hnRNPA2 LC and FUS LC. Conditions: 20 μM proteins (~1% fluorescently labeled), 20 mM MES pH 5.5, 50 mM NaCl, 150 mM urea. Scale bar: 10 μm.

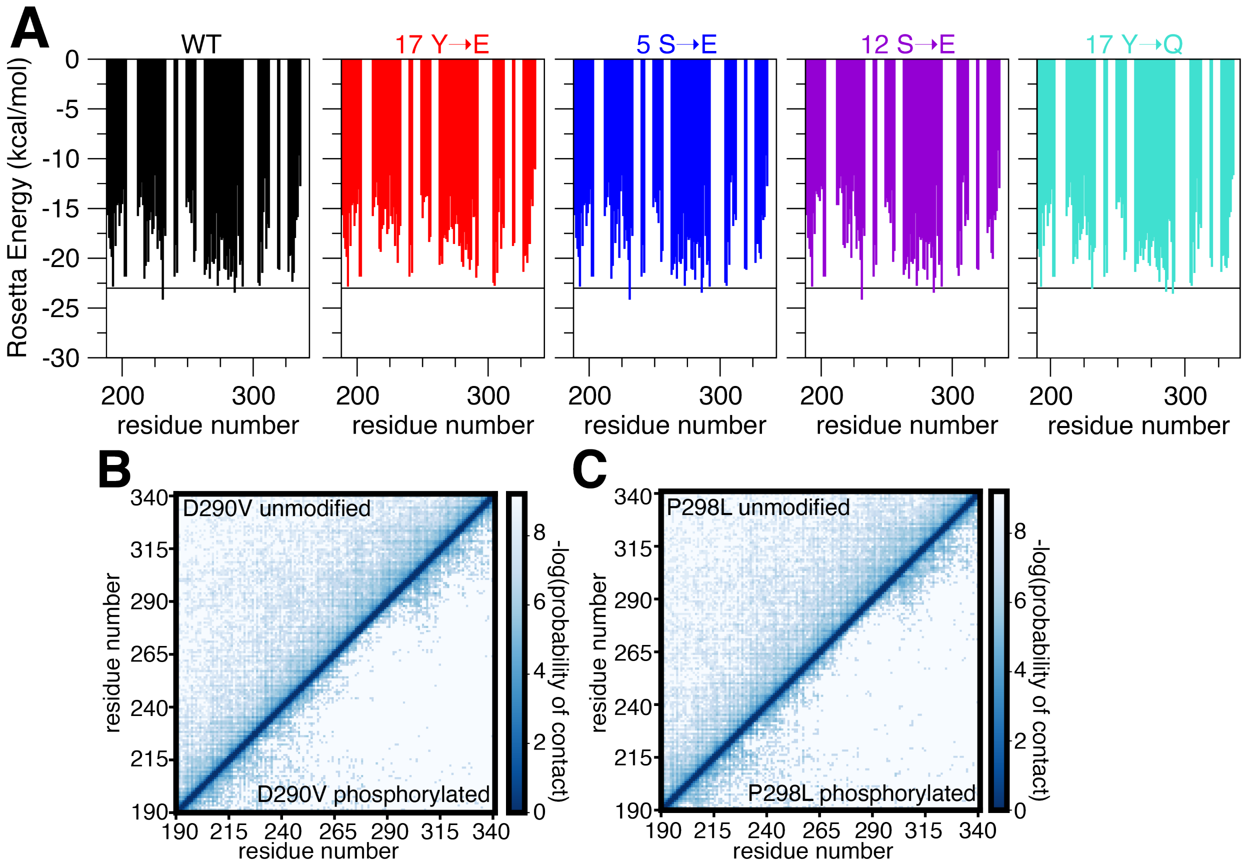

Appendix Figure S9: Phosphorylation reduces prion-like character of hnRNPA2. Related to Figure 4.

A) ZipperDB analysis (Thompson et al., 2006) of the LC domains of the same sequences as in Figure 4B shows decreased Rosetta energy steric zipper forming hexapeptides in the 17 Y→E mutant, but not in 5 S→E, 12 S→E, or 17 Y→Q.

B-C) Coarse grained simulations of D290V **(B)** and P298L **(C)** show that compared to the unphosphorylated form, phosphotyrosine hnRNPA2 LC forms fewer contacts with itself.

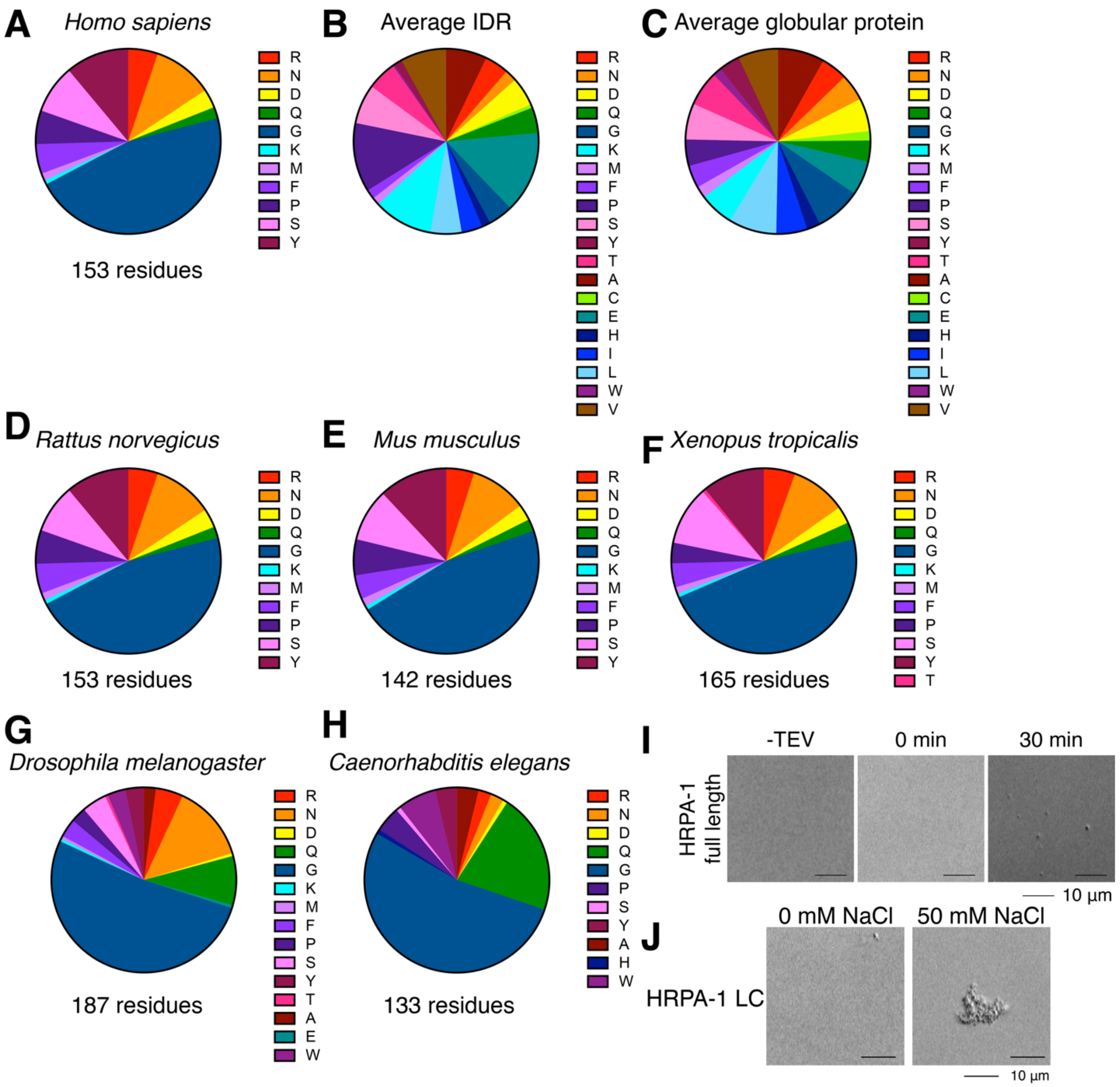

Appendix Figure S10: hnRNPA2 LC sequence composition and ability to self-assemble is conserved. Related to Figure 5.

A-H) Pie charts of hnRNPA2 LC and ortholog sequence composition. **A)** *Homo sapiens* hnRNPA2 LC is predominantly glycine, while **B)** the average IDR (Tompa, 2002) and **C)** average globular protein (Tompa, 2002) have much greater variation in sequence composition. Vertebrates **D)** *Rattus novegicus*, **E)** *Mus musculus*, and **F)** *Xenopus tropicalis* all show similar hnRNPA2 LC sequence composition to *Homo sapiens*. Invertebrates **G)** *Drosophila* and **H)** *C. elegans* have a similarly high glycine content in the LC of their hnRNPA2 orthologs.

I) Recombinant HRPA-1 FL is capable of LLPS after cleavage of a maltose binding protein solubility tag. Conditions: 20 µM protein, 20 mM Tris pH 7.4, 50 mM NaCl. Scale bar: 10 µm.

J) Recombinant HRPA-1 LC is soluble at 0 mM NaCl but forms amorphous aggregates in 50 mM NaCl. Conditions: 20 µM protein, 20 mM MES pH 5.5 150 mM urea, salt concentration as indicated. Scale bar: 10 µm

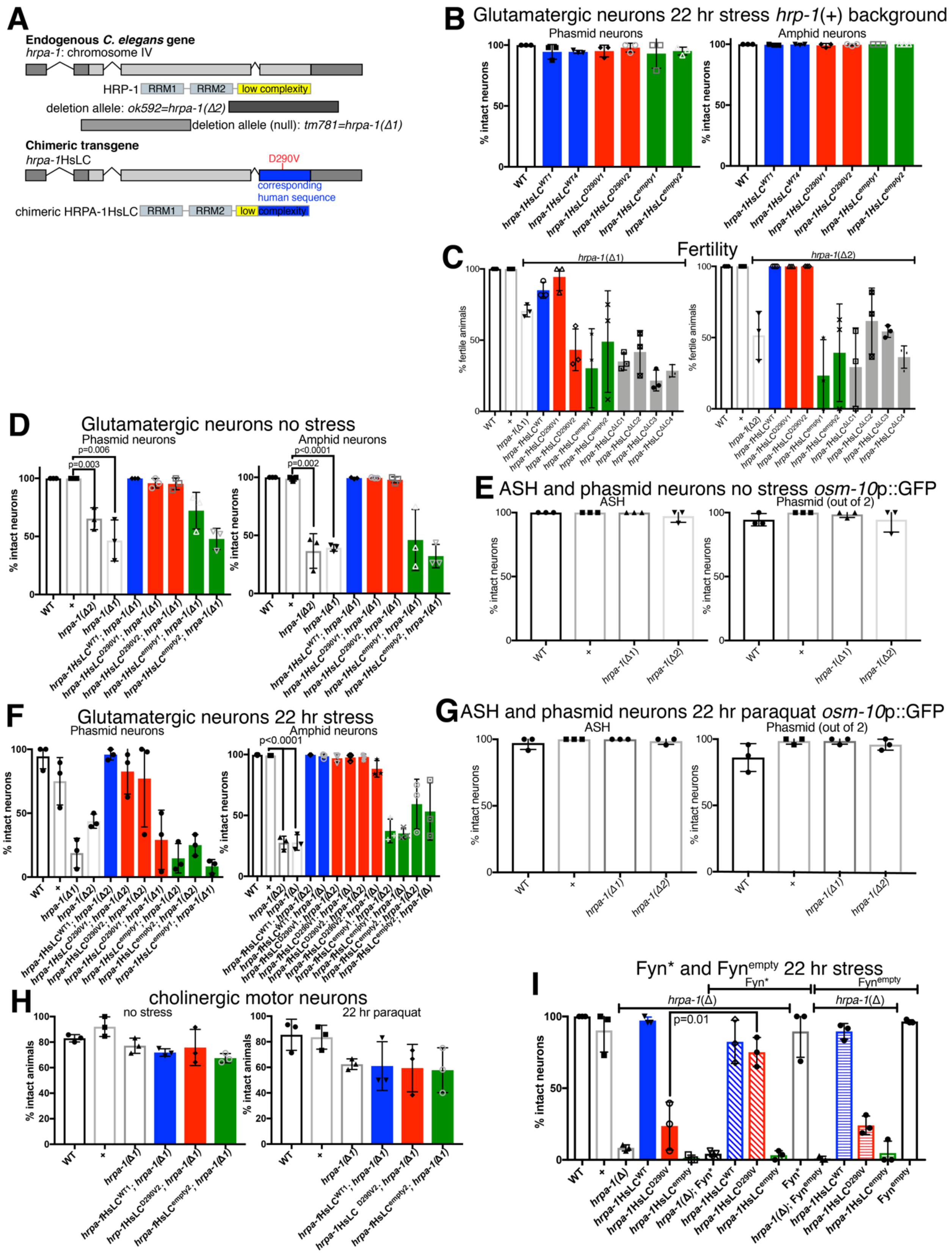
A
Endogenous C. elegans gene
hrpa-1: chromosome IV
HRP-1 RRM1 RRM2 low complexity
deletion allele: ok592=hrpa-1(Δ2)
deletion allele (null): tm781=hrpa-1(Δ1)
Chimeric transgene
hrpa-1HsLC
D290V
corresponding human sequence
chimeric HRPA-1HsLC RRM1 RRM2 low complexity
B Glutamatergic neurons 22 hr stress hrp-1(+) background
Phasmid neurons
Amphid neurons
% intact neurons
C Fertility
hrpa-1(Δ1)
hrpa-1(Δ2)
% fertile animals
D Glutamatergic neurons no stress
Phasmid neurons
Amphid neurons
p=0.006
p=0.003
p<0.0001
p=0.002
E ASH and phasmid neurons no stress osm-10p::GFP
ASH
Phasmid (out of 2)
F Glutamatergic neurons 22 hr stress
Phasmid neurons
Amphid neurons
p<0.0001
G ASH and phasmid neurons 22 hr paraquat osm-10p::GFP
ASH
Phasmid (out of 2)
H cholinergic motor neurons
no stress
22 hr paraquat
% intact animals
I Fyn* and Fynempty 22 hr stress
Fyn*
Fynempty
hrpa-1(Δ)
p=0.01

Appendix Figure S11: *hrpa-1*HsLC^{D290V} animals show glutamatergic neurodegeneration rescued by expression of Fyn kinase. Related to Figure 6, see also Tables S1, Table S2, and Figure S12.

A) Schematic depicting *hrpa-1* gene duplicated from Figure 6A with addition of *hrpa-1(ok592)* deletion allele.

B) With 22 hours paraquat stress on an *hrpa-1(+)* background, *hrpa-1*HsLC^{WT}, *hrpa-1*HsLC^{D290V}, or *hrpa-1*HsLC^{empty} animals do not have any amphid or phasmid neuron degeneration. N=12 animals/genotype/trial, 3 trials.

C) Animals carrying an *hrpa-1(Δ)* allele (*ok592* = 2, *tm781* = 1) do not always lay eggs, thus indicating they have fertility and/or vulval defects. These defects are not rescued by expression of *hrpa-1*HsLC$^{\Delta LC}$ or *hrpa-1*HsLC^{empty} transgenes. On the *hrpa-1*(*ok592*) background, expression of *hrpa-1*HsLC^{WT} or *hrpa-1*HsLC^{D290V} fully rescued the fertility defect to normal levels. On the *hrpa-1*(*tm781*) background, expression of *hrpa-1*HsLC^{WT} or *hrpa-1*HsLC^{D290V1} transgenes improved fertility, while *hrpa-1*HsLC^{empty} or *hrpa-1*HsLC^{D290V2} did not, possibly because those animals have multiple protruding vulva (pVul and Muv), which can hamper egg laying. N=12 animals/genotype/trial, 3 trials.

D) Without exposure to stress, both deletion alleles have milder dye filling defects in both amphid and phasmid neurons, but these defects are fully rescued by expression of *hrpa-1*HsLC^{WT} or *hrpa-1*HsLC^{D290V}. N=10-12 animals/genotype/trial, 3 trials.

E) Without stress, there is no difference in the number of *osm-10*p::GFP expressing neurons (1 ASH neuron per side in the head, 1 phasmid neuron per side in the tail) between WT and either *hrpa-1*(Δ) allele, indicating that the neurons are degenerating, not dying. ASH is a glutamatergic sensory neuron with exposed cilia in the head. N=12 animals/genotype/trial, 3 trials.

F) Both *hrpa-1* deletion alleles (*hrpa-1(Δ1) = hrpa-1(tm781), hrpa-1(Δ2) = hrpa-1(ok592)*) have substantial head and tail glutamatergic neuron degeneration after 22 hours of paraquat stress. *hrpa-1(tm781)* has more phasmid neuron degeneration than *hrpa-1(ok592)* (which likely expresses functional RNA binding domains) both by itself and with expression of *hrpa-1*HsLC^{D290V}. *hrpa-1*HsLC^{D290V} fully rescues the amphid neuron degeneration of both deletion alleles. N=6-12 animals/genotype/trial, 3 trials.

G) After exposure to 22 hours paraquat stress, there is no different in the number of *osm-10*p::GFP expressing neurons (1 ASH neuron per side in the head, 1 phasmid neuron per side in the tail) between WT and either *hrpa-1*(Δ) allele, indicating that the neurons are degenerating, not dying. N=12 animals/genotype/trial, 3 trials.

H) *hrpa-1(Δ), hrpa-1*HsLC^{WT}, *hrpa-1*HsLC^{D290V}, and *hrpa-1*HsLC^{empty} all have similar levels of mild cholinergic neurodegeneration both without stress and after 22 hours of paraquat induced oxidative stress. N=12-16 animals/genotype/trial, 3 trials each no stress and 22 hours paraquat. WT = *hrpa-1(+) IV; vsIs48[unc-17p::GFP],* all animals are expressing *vsIs48[unc-17p::GFP]*.

I) Independent Fyn* and Fyn^{empty} transgenes show the same trend as the initial lines; Fyn* rescues *hrpa-1*HsLC^{D290V} neurodegeneration (p=0.01) but not *hrpa-1*(Δ), while Fyn^{empty} does not rescue. N=4-12 animals/genotype/trial, 3 trials.

A

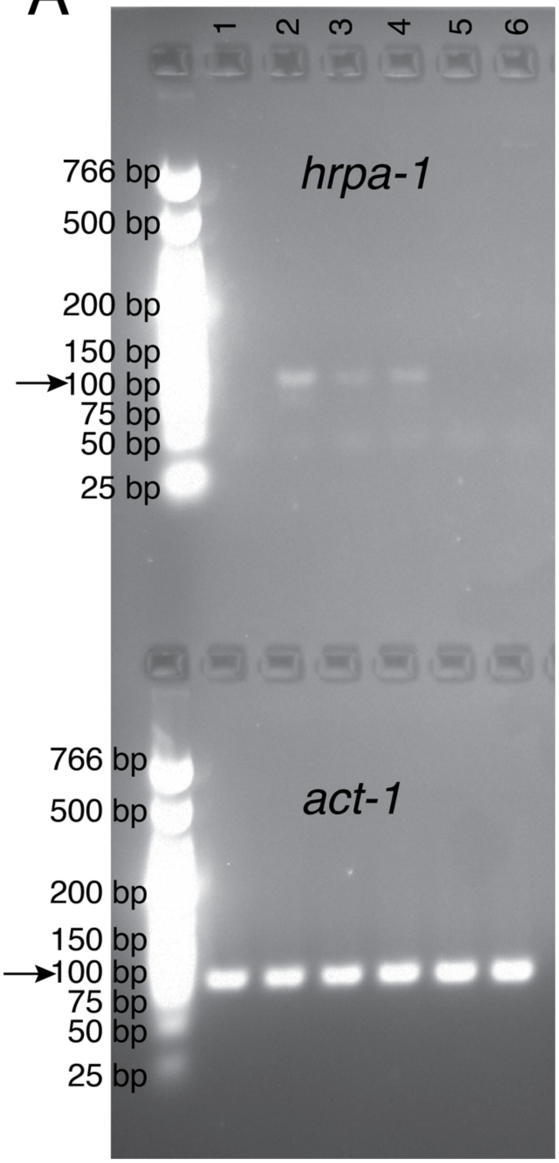

B

lane number	strain name	descriptive name
1	N2	WT
2	HA3634	*hrpa-1*HsLC^{WT}
3	HA3641	*hrpa-1*HsLC^{D290V}
4	HA3642	*hrpa-1*HsLC^{D290V}
5	HA3677	*hrpa-1*HsLC^{empty}
6	HA3697	*hrpa-1*HsLC^{empty}

Appendix Figure S12: Expression of transgenes in *C. elegans*. Related to Figures 5, 6, S11, and Table S1.

A) RT-PCR of animals carrying integrated arrays transcribing chimeric *hrpa-1* (lanes B 2 – B 4) express chimeric *hrpa-1* while WT animals and *hrpa-1*HsLC^{empty} animals do not (top). All animals tested express actin from the endogenous act-1 gene.

B) Table indicating strain used for RT-PCR experiments, corresponding lane, and the descriptive name of the strain used in the text.

Appendix Table S1: Table of *C. elegans* strains used in this study.

Strain Name	Genotype	Figures
N2	*hrpa-1(+) IV*	**6**B, D, E, G, H, **S6**B, C, D, E, G, H (WT)
GE24	*pha-1(e2123) III*	
HA3895	*rtEx986[mec-4p::hrpa-1HsLCWTmScarlet::hrpa-1 3'UTR + pBX + mec-4p::GFP + salmon sperm DNA]; pha-1(e2123) III*	**5**E (3)
HA3897	*rtEx988[mec-4p::hrpa-1HsLCWTmScarlet::hrpa-1 3'UTR + pBX + mec-4p::GFP + salmon sperm DNA]; pha-1(e2123) III*	**5**E (4)
HA3922	*rtEx989[mec-4p::hrpa-1mScarlet::hrpa-1 3'UTR + pBX + mec-4p::GFP + salmon sperm DNA]; pha-1(e2123) III*	**5**E (1)
HA3923	*rtEx990[mec-4p::hrpa-1mScarlet::hrpa-1 3'UTR + pBX + mec-4p::GFP + salmon sperm DNA]; pha-1(e2123) III*	**5**E (2)
HA3928	*rtEx995[mec-4p::hrpa-1HsLCWTmScarlet::hrpa-1 3'UTR + mec-4p::Fyn^{empty}::unc-54 3'UTR + pBX + mec-4p::GFP + salmon sperm DNA]; pha-1(e2123) III*	**5**F, G (5)
HA3929	*rtEx996[mec-4p::hrpa-1HsLCWTmScarlet::hrpa-1 3'UTR + mec-4p::Fyn^{empty}::unc-54 3'UTR + pBX + mec-4p::GFP + salmon sperm DNA]; pha-1(e2123) III*	**5**F, G (6)
HA3931	*rtEx998[mec-4p::hrpa-1HsLCD290VmScarlet::hrpa-1 3'UTR + mec-4p::Fyn^{empty}::unc-54 3'UTR + pBX + mec-4p::GFP + salmon sperm DNA]; pha-1(e2123) III*	**5**F, G (7)
HA3939	*rtEx999[mec-4p::hrpa-1HsLCD290VmScarlet::hrpa-1 3'UTR + mec-4p::Fyn^{empty}::unc-54 3'UTR + pBX + mec-4p::GFP + salmon sperm DNA]; pha-1(e2123) III*	**5**F, G (8)
HA3940	*rtEx1000[mec-4p::hrpa-1HsLCWTmScarlet::hrpa-1 3'UTR + mec-4p::FynY531F::unc-54 3'UTR + pBX + mec-4p::GFP + salmon sperm DNA]; pha-1(e2123) III*	**5**F, G (1)
HA3941	*rtEx1001[mec-4p::hrpa-1HsLCWTmScarlet::hrpa-1 3'UTR + mec-4p::FynY531F::unc-54 3'UTR + pBX + mec-4p::GFP + salmon sperm DNA]; pha-1(e2123) III*	**5**F, G (2)
HA3942	*rtEx1002[mec-4p::hrpa-1HsLCD290VmScarlet::hrpa-1 3'UTR + mec-4p::FynY531F::unc-54 3'UTR + pBX + mec-4p::GFP + salmon sperm DNA]; pha-1(e2123) III*	**5**F, G (3)
HA3943	*rtEx1003[mec-4p::hrpa-1HsLCD290VmScarlet::hrpa-1 3'UTR + mec-4p::FynY531F::unc-54 3'UTR + pBX + mec-4p::GFP + salmon sperm DNA]; pha-1(e2123) III*	**5**F, G (4)
HA3	*osm-10p::GFP (rtIs11)*	**S6**F (WT)
HA3649	*tmC25[tmIs1241]/+ IV; osm-10p::GFP*	**S6**F (+)
HA3650	*hrpa-1(ok592)/tmC25[tmIs1241] IV; osm-10p::GFP*	**S6**F (*hrpa-1*Δ2)
HA3651	*hrpa-1(tm781)/tmC25[tmIs1241] IV; osm-10p::GFP*	**S6**F (*hrpa-1*Δ1)
HA3608	*tmC25[tmIs1241]/+ IV*	**6**B, D, E, G, H, **S6**B, D, E, H (+)
HA3438	*hrpa-1(ok592)/tmC25[tmIs1241] IV*	**S6**B, D, E, (*hrpa-1*Δ2)
HA3450	*hrpa-1(tm781)/tmC25[tmIs1241] IV*	**6**B, D, E, G, H, **S6**H (*hrpa-1*Δ)

		**S6**B, D, E, (*hrpa-1*Δ1)
HA3634	*rtIs83[hrpa-1p::hrpa-1HsLC*^{WT}*::hrpa-1 3'UTR + elt-2p::GFP + salmon sperm DNA]*	**S6**C (*hrpa-1*HsLC^{WT1})
HA3635	*rtIs84[hrpa-1p::hrpa-1HsLC*^{WT}*::hrpa-1 3'UTR + elt-2p::GFP + salmon sperm DNA]*	**S6**C (*hrpa-1*HsLC^{WT4})
HA3641	*rtIs81[hrpa-1p::hrpa-1HsLC*^{D290V1}*::hrpa-1 3'UTR + elt-2p::GFP + salmon sperm DNA]*	**S6**C (*hrpa-1*HsLC^{D290V1})
HA3642	*rtIs82[hrpa-1p::hrpa-1HsLC*^{D290V2}*::hrpa-1 3'UTR + elt-2p::GFP + salmon sperm DNA]*	**S6**C (*hrpa-1*HsLC^{D290V2})
HA3677	*rtIs93[hrpa-1p::hrpa-1HsLC*^{empty1}*::hrpa-1 3'UTR + elt-2::GFP + salmon sperm DNA]*	**S6**C (*hrpa-1*HsLC^{empty1})
HA3697	*rtIs94[hrpa-1p::hrpa-1HsLC*^{empty2}*::hrpa-1 3'UTR + elt-2::GFP + salmon sperm DNA]*	**S6**C (*hrpa-1*HsLC^{empty2})
HA3684	*rtIs77[hrpa-1p::hrpa-1HsLC*$^{\Delta LC1}$*::hrpa-1 3'UTR + elt-2p::GFP + salmon sperm DNA]; hrpa-1(ok592)/tmC25[tmIs1241] IV*	**S6**B (*hrpa-1*HsLC$^{\Delta LC1}$; *hrpa-1*(Δ2))
HA3683	*rtIs77[hrpa-1p::hrpa-1HsLC*$^{\Delta LC1}$*::hrpa-1 3'UTR + elt-2p::GFP + salmon sperm DNA]; hrpa-1(tm781)/tmC25[tmIs1241] IV*	**S6**B (*hrpa-1*HsLC$^{\Delta LC1}$; *hrpa-1*(Δ1))
HA3682	*rtIs78[hrpa-1p::hrpa-1HsLC*$^{\Delta LC2}$*::hrpa-1 3'UTR + elt-2p::GFP + salmon sperm DNA]; hrpa-1(ok592)/tmC25[tmIs1241] IV*	**S6**B (*hrpa-1*HsLC$^{\Delta LC2}$; *hrpa-1*(Δ2))
HA3686	*rtIs78[hrpa-1p::hrpa-1HsLC*$^{\Delta LC2}$*::hrpa-1 3'UTR + elt-2p::GFP + salmon sperm DNA]; hrpa-1(tm781)/tmC25[tmIs1241] IV*	**S6**B (*hrpa-1*HsLC$^{\Delta LC2}$; *hrpa-1*(Δ1))
HA3690	*rtIs79[hrpa-1p::hrpa-1HsLC*$^{\Delta LC3}$*::hrpa-1 3'UTR + elt-2p::GFP + salmon sperm DNA]; hrpa-1(ok592)/tmC25[tmIs1241] IV*	**S6**B (*hrpa-1*HsLC$^{\Delta LC3}$; *hrpa-1*(Δ2))
HA3685	*rtIs79[hrpa-1p::hrpa-1HsLC*$^{\Delta LC3}$*::hrpa-1 3'UTR + elt-2p::GFP + salmon sperm DNA]; hrpa-1(tm781)/tmC25[tmIs1241] IV*	**S6**B (*hrpa-1*HsLC$^{\Delta LC3}$; *hrpa-1*(Δ1))
HA3691	*rtIs80[hrpa-1p::hrpa-1HsLC*$^{\Delta LC4}$*::hrpa-1 3'UTR + elt-2p::GFP + salmon sperm DNA]; hrpa-1(ok592)/tmC25[tmIs1241] IV*	**S6**B (*hrpa-1*HsLC$^{\Delta LC4}$; *hrpa-1*(Δ2))
HA3692	*rtIs80[hrpa-1p::hrpa-1HsLC*$^{\Delta LC4}$*::hrpa-1 3'UTR + elt-2p::GFP + salmon sperm DNA]; hrpa-1(tm781)/tmC25[tmIs1241] IV*	**S6**B (*hrpa-1*HsLC$^{\Delta LC4}$; *hrpa-1*(Δ1))
HA3654	*rtIs83[hrpa-1p::hrpa-1HsLC*^{WT}*::hrpa-1 3'UTR + elt-2p::GFP + salmon sperm DNA]; hrpa-1(ok592)/tmC25[tmIs1241] IV*	**S6**B, D, E (*hrpa-1*HsLC^{WT1}; *hrpa-1*(Δ2))
HA3655	*rtIs83[hrpa-1p::hrpa-1HsLC*^{WT}*::hrpa-1 3'UTR + elt-2p::GFP + salmon sperm DNA]; hrpa-1(tm781)/tmC25[tmIs1241] IV*	**6**B, D, E, H, S6H (*hrpa-1*HsLC^{WT}; *hrpa-1*(Δ)), **S6**B, D, E (*hrpa-1*HsLC^{WT1}; *hrpa-1*(Δ1))
HA3656	*rtIs81[hrpa-1p::hrpa-1HsLC*^{D290V1}*::hrpa-1 3'UTR + elt-2p::GFP + salmon sperm DNA]; hrpa-1(ok592)/tmC25[tmIs1241] IV*	**S6**B, D, E (*hrpa-1*HsLC^{D290V1}; *hrpa-1*(Δ2))

HA3657	*rtIs81[hrpa-1p::hrpa-1HsLC^{D290V1}::hrpa-1 3'UTR* + *elt-2p::GFP* + *salmon sperm DNA]; hrpa-1(tm781)/tmC25[tmIs1241] IV*	**S6**B, D, E (*hrpa-1*HsLC^{D290V1}; *hrpa-1*(Δ1))
HA3658	*rtIs82[hrpa-1p::hrpa-1HsLC^{D290V2}::hrpa-1 3'UTR* + *elt-2p::GFP* + *salmon sperm DNA]; hrpa-1(ok592)/tmC25[tmIs1241] IV*	**S6**B, D, E (*hrpa-1*HsLC^{D290V2}; *hrpa-1*(Δ2))
HA3659	*rtIs82[hrpa-1p::hrpa-1HsLC^{D290V2}::hrpa-1 3'UTR* + *elt-2p::GFP* + *salmon sperm DNA]; hrpa-1(tm781)/tmC25[tmIs1241] IV*	**6**B, D, E, G H, S6H (*hrpa-1*HsLC^{D290V}; *hrpa-1*(Δ)), **S6**B, D, E (*hrpa-1*HsLC^{D290V2}; *hrpa-1*(Δ1))
HA3707	*rtIs93[hrpa-1p::hrpa-1HsLC^{empty1}::hrpa-1 3'UTR* + *elt-2::GFP* + *salmon sperm DNA]; hrpa-1(ok592)/tmC25[tmIs1241] IV*	**S6**B, D, E (*hrpa-1*HsLC^{empty1}; *hrpa-1*(Δ2))
HA3708	*rtIs93[hrpa-1p::hrpa-1HsLC^{empty1}::hrpa-1 3'UTR* + *elt-2::GFP* + *salmon sperm DNA]; hrpa-1(tm781)/tmC25[tmIs1241] IV*	**S6**B, D, E (*hrpa-1*HsLC^{empty1}; *hrpa-1*(Δ1))
HA3709	*rtIs94[hrpa-1p::hrpa-1HsLC^{empty2}::hrpa-1 3'UTR* + *elt-2::GFP* + *salmon sperm DNA]; hrpa-1(ok592)/tmC25[tmIs1241] IV*	**S6**B, D, E (*hrpa-1*HsLC^{empty2}; *hrpa-1*(Δ2))
HA3710	*rtIs94[hrpa-1p::hrpa-1HsLC^{empty2}::hrpa-1 3'UTR* + *elt-2::GFP* + *salmon sperm DNA]; hrpa-1(tm781)/tmC25[tmIs1241] IV*	**6**B, D, E, G, H, S6H (*hrpa-1*HsLC^{empty}; *hrpa-1*(Δ)), **S6**B, D, E (*hrpa-1*HsLC^{empty2}; *hrpa-1*(Δ1))
LX929	*hrpa-1(+) IV; vsIs48 [unc-17p::GFP]*	**S6**G (WT)
HA3877	*tmC25[tmIs1241] IV; vsIs48 [unc-17p::GFP]*	**S6**G (+)
HA3491	*hrpa-1(tm781)/tmC25[tmIs1241] IV;vsIs48 [unc-17p::GFP]*	**S6**G (*hrpa-1*(Δ1))
HA3742	*rtIs83[hrpa-1p::hrpa-1HsLC^{WT}::hrpa-1 3'UTR* + *elt-2p::GFP* + *salmon sperm DNA]; hrpa-1(tm781)/tmC25[tmIs1241] IV; vsIs48 [unc-17p::GFP]*	**S6**G (*hrpa-1*HsLC^{WT1}; *hrpa-1*(Δ1))
HA3744	*rtIs82[hrpa-1p::hrpa-1HsLC^{D290V2}::hrpa-1 3'UTR* + *elt-2p::GFP* + *salmon sperm DNA]; hrpa-1(tm781)/tmC25[tmIs1241] IV; vsIs48 [unc-17p::GFP]*	**S6**G (*hrpa-1*HsLC^{D290V2}; *hrpa-1*(Δ1))
HA3746	*rtIs94[hrpa-1p::hrpa-1HsLC^{empty2}::hrpa-1 3'UTR* + *elt-2::GFP* + *salmon sperm DNA]; hrpa-1(tm781)/tmC25[tmIs1241] IV; vsIs48 [unc-17p::GFP]*	**S6**G (*hrpa-1*HsLC^{empty2}; *hrpa-1*(Δ1))
HA3703	*tdp-1(tgx58) II*	**6**D (*tdp-1*(Δ))

HA3760	*tdp-1(tgx58) II; hrpa-1(tm781)/tmC25[tmIs1241] IV*	**6**D (*hrpa-1*(Δ); *tdp-1*(Δ))
HA3763	*rtIs94[hrpa-1p::hrpa-1HsLC*^{empty2}*::hrpa-1 3'UTR + elt-2::GFP + salmon sperm DNA]; hrpa-1(tm781)/tmC25[tmIs1241] IV; tdp-1(tgx58) II*	**6**D (*hrpa-1*HsLC^{empty}; *hrpa-1*(Δ); *tdp-1*(Δ))
HA3764	*rtIs83[hrpa-1p::hrpa-1HsLC*^{WT}*::hrpa-1 3'UTR + elt-2p::GFP + salmon sperm DNA]; hrpa-1(tm781)/tmC25[tmIs1241] IV; tdp-1(tgx58) II*	**6**D (*hrpa-1*HsLC^{WT}; *hrpa-1*(Δ); *tdp-1*(Δ))
HA3765	*rtIs82[hrpa-1p::hrpa-1HsLC*^{D290V2}*::hrpa-1 3'UTR + elt-2p::GFP + salmon sperm DNA]; hrpa-1(tm781)/tmC25[tmIs1241] IV; tdp-1(tgx58) II*	**6**D (*hrpa-1*HsLC^{D290V}; *hrpa-1*(Δ); *tdp-1*(Δ))
HA2693	*tdp-1(ok803)*	**6**E (*tdp-1*(Δ))
HA3829	*tdp-1(ok803) II; hrpa-1(tm781)/tmC25[tmIs1241] IV*	**6**E (*hrpa-1*(Δ); *tdp-1*(Δ))
HA3833	*rtIs94[hrpa-1p::hrpa-1HsLC*^{empty2}*::hrpa-1 3'UTR + elt-2::GFP + salmon sperm DNA]; hrpa-1(tm781)/tmC25[tmIs1241] IV; tdp-1(ok803) II*	**6**E (*hrpa-1*HsLC^{empty}; *hrpa-1*(Δ); *tdp-1*(Δ))
HA3830	*rtIs83[hrpa-1p::hrpa-1HsLC*^{WT}*::hrpa-1 3'UTR + elt-2p::GFP + salmon sperm DNA]; hrpa-1(tm781)/tmC25[tmIs1241] IV; tdp-1(ok803) II*	**6**E (*hrpa-1*HsLC^{WT}; *hrpa-1*(Δ); *tdp-1*(Δ))
HA3832	*rtIs82[hrpa-1p::hrpa-1HsLC*^{D290V2}*::hrpa-1 3'UTR + elt-2p::GFP + salmon sperm DNA]; hrpa-1(tm781)/tmC25[tmIs1241] IV; tdp-1(ok803) II*	**6**E (*hrpa-1*HsLC^{D290V}; *hrpa-1*(Δ); *tdp-1*(Δ))
HA3835	*pha-1(e2123) III; rtEx969[osm-10p::FynY531F::unc-54 3'UTR + myo-2::mCherry + pBX + salmon sperm DNA]*	**6**G (Fyn*)
HA3838	*rtIs83[hrpa-1p::hrpa-1HsLC*^{WT1}*::hrpa-1 3'UTR + elt-2p::GFP + salmon sperm DNA]; tm781/tmC25[tmIs1241] IV; rtEx969[osm-10p::FynY531F::unc-54 3'UTR + myo-2::mCherry + pBX + salmon sperm DNA]*	**6**G (*hrpa-1*HsLC^{WT}; *hrpa-1*(Δ); Fyn*)
HA3839	*rtIs82[hrpa-1p::hrpa-1HsLC*^{D290V2}*::hrpa-1 3'UTR + elt-2p::GFP + salmon sperm DNA]; tm781/tmC25[tmIs1241] IV; rtEx969[osm-10p::FynY531F::unc-54 3'UTR + myo-2::mCherry + pBX + salmon sperm DNA]*	**6**G (*hrpa-1*HsLC^{D290V}; *hrpa-1*(Δ); Fyn*)
HA3840	*rtIs94[hrpa-1p::hrpa-1HsLC*^{empty2}*::hrpa-1 3'UTR + elt-2p::GFP + salmon sperm DNA]; tm781/tmC25[tmIs1241] IV; rtEx969[osm-10p::FynY531F::unc-54 3'UTR + myo-2::mCherry + pBX + salmon sperm DNA]*	**6**G (*hrpa-1*HsLC^{empty}; *hrpa-1*(Δ); Fyn*)
HA3837	*hrpa-1(tm781)/tmC25[tmIs1242] IV; rtEx969[osm-10p::FynY531F::unc-54 3'UTR + myo-2::mCherry + pBX + salmon sperm DNA]*	**6**G (*hrpa-1*(Δ); Fyn*)
HA3886	*pha-1(e2123) III; rtEx981[osm10p::Fyn*^{empty}*::unc-5 + pBX + PCFJ90(myo-2p::mCherry), salmon sperm]*	**6**H (Fyn^{empty})
HA3948	*rtEx981[osm10p::Fyn*^{empty}*::unc-5 + pBX + myo-2p::mCherry, salmon sperm]; tm781/tmC25[tmIs1241] IV*	**6**H (*hrpa-1*(Δ); Fyn^{empty})
HA3949	*rtIs83[hrpa-1p::hrpa-1HsLC*^{WT1}*::hrpa-1 3'UTR + elt-2p::GFP + salmon sperm DNA]; tm781/tmC25[tmIs1241] IV; rtEx981[osm-*	**6**H (*hrpa-1*HsLC^{WT};

	10p:: Fyn^empty::unc-54 3'UTR + myo-2::mCherry + pBX + salmon sperm DNA]	*hrpa-1*(Δ); Fyn^{empty})
HA3950	*rtIs82[hrpa-1p::hrpa-1HsLC^{D290V2}::hrpa-1 3'UTR + elt-2p::GFP + salmon sperm DNA]; tm781/tmC25[tmIs1241] IV; rtEx981[osm-10p:: Fyn^{empty}::unc-54 3'UTR + myo-2::mCherry + pBX + salmon sperm DNA]*	**6**H (*hrpa-1*HsLC^{D290V}; *hrpa-1*(Δ); Fyn^{empty})
HA3951	*rtIs94[hrpa-1p::hrpa-1HsLC^{empty2}::hrpa-1 3'UTR + elt-2p::GFP + salmon sperm DNA]; tm781/tmC25[tmIs1241] IV; rtEx981[osm-10p:: Fyn^{empty}::unc-54 3'UTR + myo-2::mCherry + pBX + salmon sperm DNA]*	**6**H (*hrpa-1*HsLC^{empty}; *hrpa-1*(Δ); Fyn^{empty})
HA3890	*pha-1(e2123) III; rtEx985[osm-10p::FynY531F::unc-54 3'UTR + myo-2::mCherry + pBX + salmon sperm DNA]*	**S6**H (Fyn*)
HA3956	*hrpa-1(tm781)/tmC25[tmIs1242] IV; rtEx985[osm-10p::FynY531F::unc-54 3'UTR + myo-2::mCherry + pBX + salmon sperm DNA]*	**S6**H (*hrpa-1*(Δ); Fyn*)
HA3957	*rtIs83[hrpa-1p::hrpa-1HsLC^{WT1}::hrpa-1 3'UTR + elt-2p::GFP + salmon sperm DNA]; tm781/tmC25[tmIs1241] IV; rtEx985[osm-10p::FynY531F::unc-54 3'UTR + myo-2::mCherry + pBX + salmon sperm DNA]*	**S6**H (*hrpa-1*HsLC^{WT}; *hrpa-1*(Δ); Fyn*)
HA3958	*rtIs82[hrpa-1p::hrpa-1HsLC^{D290V2}::hrpa-1 3'UTR + elt-2p::GFP + salmon sperm DNA]; tm781/tmC25[tmIs1241] IV; rtEx985[osm-10p::FynY531F::unc-54 3'UTR + myo-2::mCherry + pBX + salmon sperm DNA]*	**S6**H (*hrpa-1*HsLC^{D290V}; *hrpa-1*(Δ); Fyn*)
HA3959	*rtIs94[hrpa-1p::hrpa-1HsLC^{empty2}::hrpa-1 3'UTR + elt-2p::GFP + salmon sperm DNA]; tm781/tmC25[tmIs1241] IV; rtEx985[osm-10p::FynY531F::unc-54 3'UTR + myo-2::mCherry + pBX + salmon sperm DNA]*	**S6**H (*hrpa-1*HsLC^{empty}; *hrpa-1*(Δ); Fyn*)
HA3889	*pha-1(e2123) III; rtEx984[osm-10p:: Fyn^{empty}::unc-54 3'UTR + myo-2::mCherry + pBX + salmon sperm DNA]*	**S6**H (Fyn^{empty})
HA3952	*hrpa-1(tm781)/tmC25[tmIs1242] IV; rtEx984[osm-10p:: Fyn^{empty}::unc-54 3'UTR + myo-2::mCherry + pBX + salmon sperm DNA]*	**S6**H (*hrpa-1*(Δ); Fyn^{empty})
HA3953	*rtIs83[hrpa-1p::hrpa-1HsLC^{WT1}::hrpa-1 3'UTR + elt-2p::GFP + salmon sperm DNA]; tm781/tmC25[tmIs1241] IV; rtEx984[osm-10p:: Fyn^{empty}::unc-54 3'UTR + myo-2::mCherry + pBX + salmon sperm DNA]*	**S6**H (*hrpa-1*HsLC^{WT}; *hrpa-1*(Δ); Fyn^{empty})
HA3954	*rtIs82[hrpa-1p::hrpa-1HsLC^{D290V2}::hrpa-1 3'UTR + elt-2p::GFP + salmon sperm DNA]; tm781/tmC25[tmIs1241] IV; rtEx984[osm-10p:: Fyn^{empty}::unc-54 3'UTR + myo-2::mCherry + pBX + salmon sperm DNA]*	**S6**H (*hrpa-1*HsLC^{D290V}; *hrpa-1*(Δ); Fyn^{empty})
HA3955	*rtIs94[hrpa-1p::hrpa-1HsLC^{empty2}::hrpa-1 3'UTR + elt-2p::GFP + salmon sperm DNA]; tm781/tmC25[tmIs1241] IV; rtEx984[osm-10p:: Fyn^{empty}::unc-54 3'UTR + myo-2::mCherry + pBX + salmon sperm DNA]*	**S6**H (*hrpa-1*HsLC^{empty}; *hrpa-1*(Δ); Fyn^{empty})

Appendix Table S2: Protein sequences for *C. elegans* constructs

Name	Protein sequence	Key
Homo sapiens hnRNPA2	*MEREKEQFRKLFIGGLSFETTEESLRNYYEQWGKLTD CVVMRDPASKRSRGFGFVTFSSMAEVDAAMAARPHS IDGRVVEPKRAVAREESGKPGAHVTVKKLFVGGIKED TEEHHLRDYFEEYGKIDTIEIITDRQSGKKRGFGFVTFD DHDPVDKIVLQKYHTINGHNAEVRKALSRQEMQEVQS SRS*GRGGNFGFGDSRGGGGNFGPGPGSNFRGGSD GYGSGRGFGDGYNGYGGGPGGGNFGGSPGYGGGR GGYGGGGPGYGNQGGGYGGGYDNYGGGNYGSGN YN**D**FGNYNQQPSNYGPMKSGNFGGSRNMGGPYGG GNYGPGGSGGSGGYGGRSRY	Human LC (190-341) **D290V**
C. elegans HRPA-1	MTDVEIKAENGSGDASLEPENLRKIFVGGLTSNTTDDL MREFYSQFGEITDIIVMRDPTTKRSRGFGFVTFSGKTE VDAAMKQRPHIIDGKTVDPKRAVPRDDKNRSESNVST KRLYVSGVREDHTEDMLTEYFTKYGTVTKSEIILDKAT QKPRGFGFVTFDDHDSVDQCVLQKSHMVNGHRCDV RKGLSKDEMSKAQMNRDRETRGGRSRDGQRGGYN GGGGGGGGWGGPAQRGGPGAYGGPGGGGQGGYG GDYGGGWGQQGGGGQGGWGGPQQQQGGGGWG QQGGGGQGGWGGPQQQQQGGWGGPQQGGGGGG WGGQGQQQGGWGGQSGAQQWAHAQGGNRNY	*C. elegans* LC (214-346)
HRPA-1mScarlet	MTDVEIKAENGSGDASLEPENLRKIFVGGLTSNTTDDL MREFYSQFGEITDIIVMRDPTTKRSRGFGFVTFSGKTE VDAAMKQRPHIIDGKTVDPKRAVPRDDKNRSESNVST KRLYVSGVREDHTEDMLTEYFTKYGTVTKSEIILDKAT QKPRGFGFVTFDDHDSVDQCVLQKSHMVNGHRCDV RKGLSKDEMSKAQMNRDRETRGGRSRDMVSKGEAV IKEFMRFKVHMEGSMNGHEFEIEGEGEGRPYEGTQT AKLKVTKGGPLPFSWDILSPQFMYGSRAFTKHPADIP DYYKQSFPEGFKWERVMNFEDGGAVTVTQDTSLEDG TLIYKVKLRGTNFPPDGPVMQKKTMGWEASTERLYPE DGVLKGDIKMALRLKDGGRYLADFKTTYKAKKPVQMP GAYNVDRKLDITSHNEDYTVVEQYERSEGRHSTGGM DELYKGQRGGYNGGGGGGGGWGGPAQRGGPGAY GGPGGGGQGGYGGDYGGGWGQQGGGGQGGWGG PQQQQGGGGWGQQGGGGQGGWGGPQQQQQGG WGGPQQGGGGGGWGGQGQQQGGWGGQSGAQQ WAHAQGGNRNY	mScarlet *C. elegans* LC (214-346)
HRPA-1HsLC$^{WT/D290V}$ mScarlet	MTDVEIKAENGSGDASLEPENLRKIFVGGLTSNTTDDL MREFYSQFGEITDIIVMRDPTTKRSRGFGFVTFSGKTE VDAAMKQRPHIIDGKTVDPKRAVPRDDKNRSESNVST KRLYVSGVREDHTEDMLTEYFTKYGTVTKSEIILDKAT QKPRGFGFVTFDDHDSVDQCVLQKSHMVNGHR CDVRKGLSKDEMSKAQMNRDRETRGGRSRDMVSKG EAVIKEFMRFKVHMEGSMNGHEFEIEGEGEGRPYEG TQTAKLKVTKGGPLPFSWDILSPQFMYGSRAFTKHPA DIPDYYKQSFPEGFKWERVMNFEDGGAVTVTQDTSL EDGTLIYKVKLRGTNFPPDGPVMQKKTMGWEASTER LYPEDGVLKGDIKMALRLKDGGRYLADFKTTYKAKKP VQMPGAYNVDRKLDITSHNEDYTVVEQYERSEGRHS TGGMDELYKGQRGGYNGGGGGGGGWGGPAQRGG	mScarlet *C. elegans* LC (214-254) Human LC (250-341) **D290V** (human hnRNPA2 numbering)

	PGAYGGPGGGGQGGYGGDYGGGRGGYGGGGPGY GNQGGGYGGGYDNYGGGNYGSGNYN**D**FGNYNQQP SNYGPMKSGNFGGSRNMGGPYGGGNYGPGGSGGS GGYGGRSRY	
HRPA-1HsLC$^{WT/D290V}$	MTDVEIKAENGSGDASLEPENLRKIFVGGLTSNTTDDL MREFYSQFGEITDIIVMRDPTTKRSRGFGFVTFSGKTE VDAAMKQRPHIIDGKTVDPKRAVPRDDKNRSESNVST KRLYVSGVREDHTEDMLTEYFTKYGTVTKSEIILDKAT QKPRGFGFVTFDDHDSVDQCVLQKSHMVNGHRCDV RKGLSKDEMSKAQMNRDRETRGGRSRDGQRGGYN GGGGGGGGWGGPAQRGGPGAYGGPGGGGQGGYG GDYGGGRGGYGGGGPGYGNQGGGYGGGYDNYGG GNYGSGNYN**D**FGNYNQQPSNYGPMKSGNFGGSRN MGGPYGGGNYGPGGSGGSGGYGGRSRY	*C. elegans* LC (214-254) Human LC (250-341) **D290V** (human hnRNPA2 numbering)
HRPA-1HsLC$^{\Delta LC}$	MTDVEIKAENGSGDASLEPENLRKIFVGGLTSNTTDDL MREFYSQFGEITDIIVMRDPTTKRSRGFGFVTFSGKTE VDAAMKQRPHIIDGKTVDPKRAVPRDDKNRSESNVST KRLYVSGVREDHTEDMLTEYFTKYGTVTKSEIILDKAT QKPRGFGFVTFDDHDSVDQCVLQKSHMVNGHRCDV RKGLSKDEMSKAQMNRDRETRGGRSRDGQRGGYN GGGGGGGGWGGPAQRGGPGAYGGPGGGGQGGYG GDYGGGR	*C. elegans* LC (214-254)
